# Supplementary figures and images for: Comprehensive profiling of translation initiation in influenza virus infected cells
Source: PLoS Pathog. 2019 Jan 23;15(1):e1007518. doi: 10.1371/journal.ppat.1007518 (PMC6361465; doi:10.1371/journal.ppat.1007518)

**A**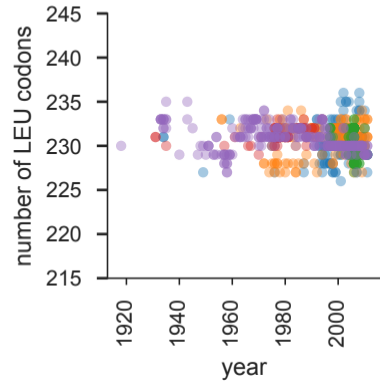**B**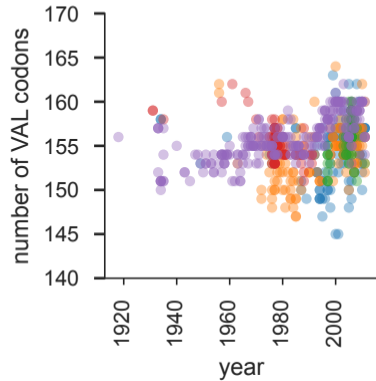**C**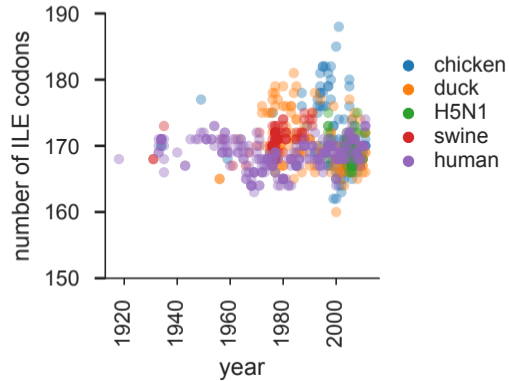

Supplement: S1 Fig — The number of LEU, VAL, ILE codons in reading frames 0 of the influenza genome over time in human, avian, and swine lineages. There is no systematic trend for enrichment or depletion of any of the amino acids. (PDF) [file ppat.1007518.s001.pdf]

**A**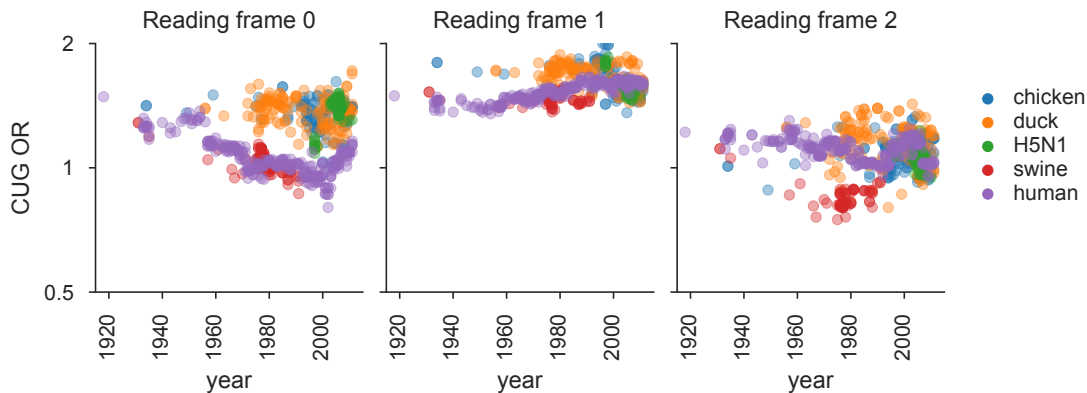**B**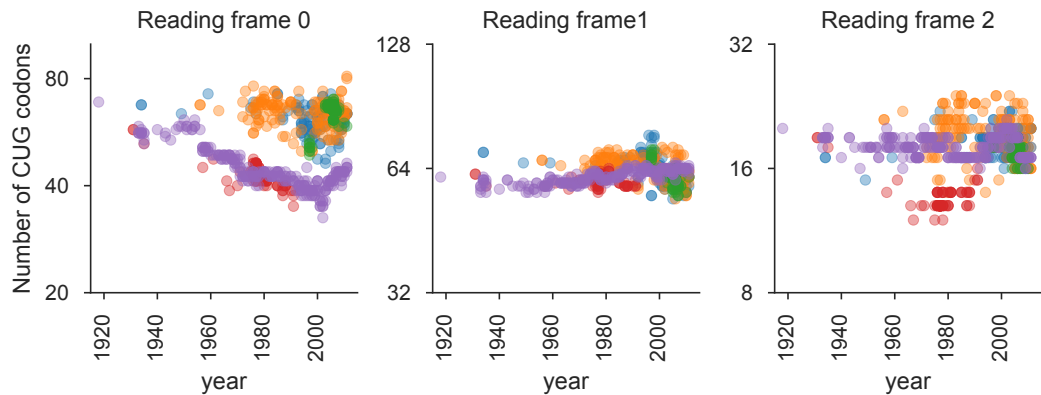

Supplement: S2 Fig — (A) Evolution of the CUG odds ratio (CUGC×U×G) in reading frames 0, 1, and 2 over time in human, avian, and swine lineages. The selection against CUG in reading frame 0 exists even when we use the odds ratio to correct for nucleotide usage. (B) Evolution of the number of CUG codons in reading frames 0, 1 and 2 of the influenza genome over time in human, avian, and swine lineages. (PDF) [file ppat.1007518.s002.pdf]

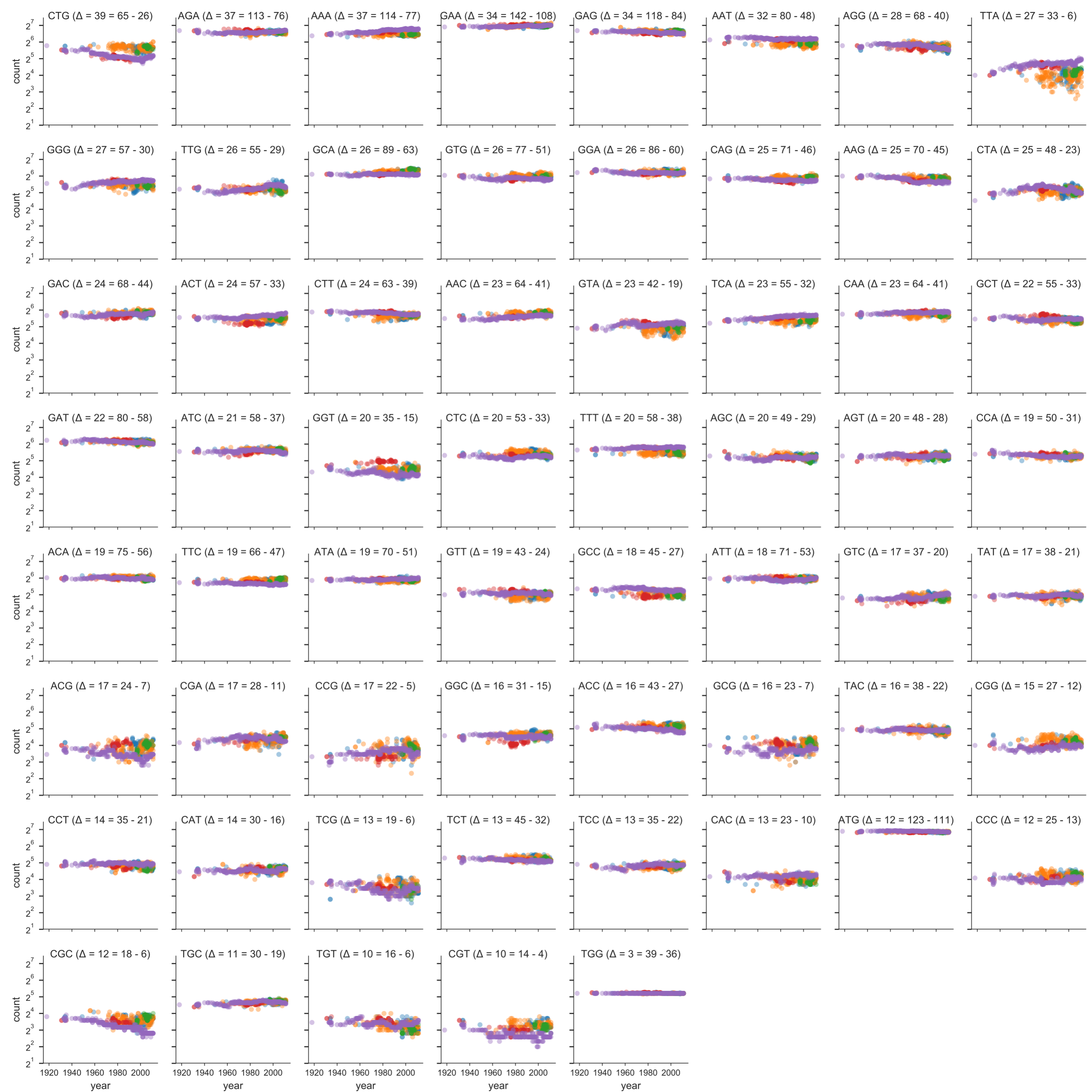

Supplement: S3 Fig — The numbers of all 61 non-stop codons in the influenza genome in reading frame 0 over time in human, avian, and swine lineages. This figure shows data comparable to Fig 1A but for all codons, and the points are colored according to the same legend used in that figure. The codons are sorted in this plot by the maximal change in codon number between any two plotted viral isolates, and the magnitude of this maximal change for each codon is indicated in the plot title. As can be seen, CUG has the largest maximal change in the number of times in which it appears. (PDF) [file ppat.1007518.s003.pdf]

# CUG start sites from Ingolia et al

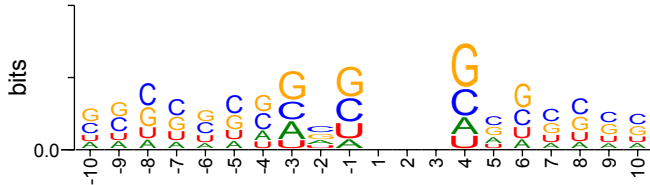

Supplement: S4 Fig — The consensus CUG start site motif was calculated using the Ingolia ribosome profiling dataset [27]. The most prominent feature of the consensus is a G at the +4 position. (PDF) [file ppat.1007518.s004.pdf]

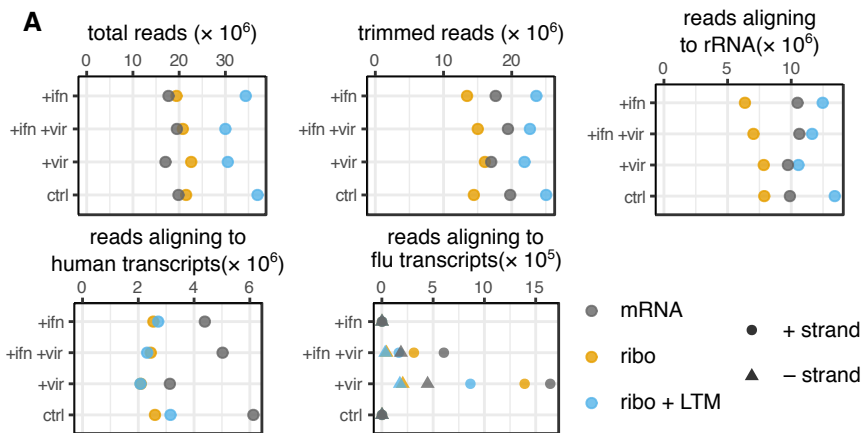

**B**

— ribo — ribo + LTM

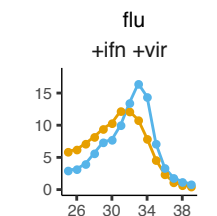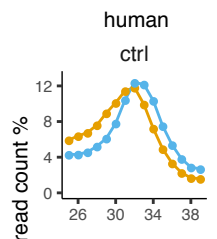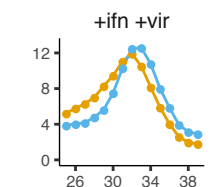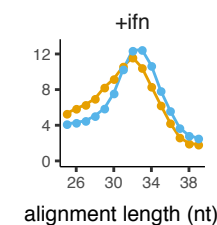

**C**

— ribo — ribo + LTM

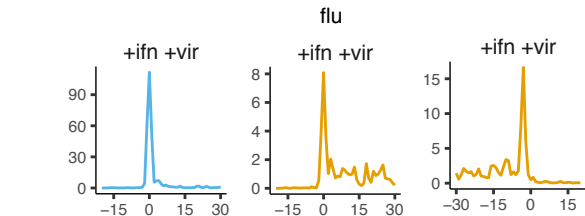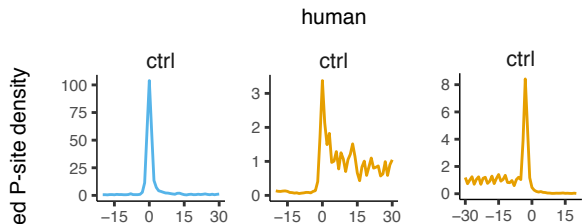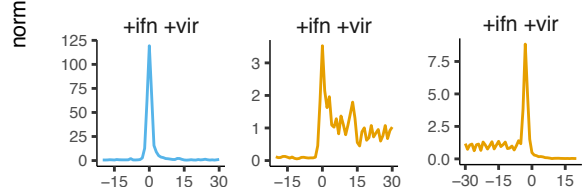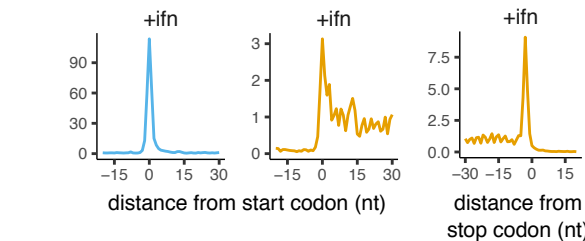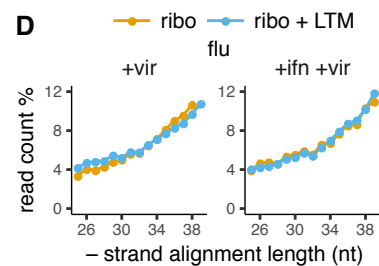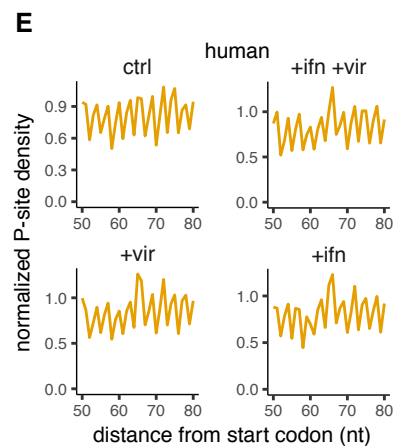

**F**

— mRNA — ribo

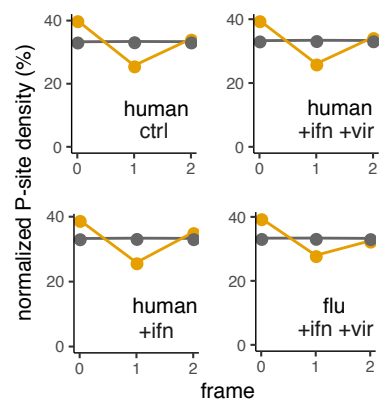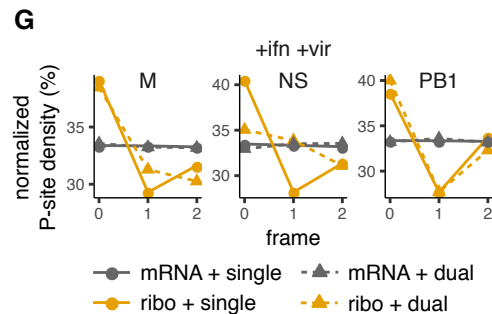

Supplement: S6 Fig — (A) Number of input, trimmed, rRNA-aligned, and human/influenza transcript-aligned reads for assays and samples shown in Fig 2. Number of reads aligning to + and—strands of influenza genome are shown separately. (B) Length distribution of viral (+ strand only) and human transcript aligned reads for Ribo-seq and Ribo-seq + LTM samples. (C) Metagene alignment of average P-site density around annotated start codons in viral and human transcripts for Ribo-seq + LTM and Ribo-seq samples. Right panels show the same for annotated stop codons for Ribo-seq samples. (D) Length distribution of viral (- strand only) genome aligned reads for Ribo-seq and Ribo-seq + LTM samples. (E) Metagene P-site density showing 3 nucleotide periodicity in a representative region of human transcripts for Ribo-seq samples. (F) Normalized P-site density in each of the reading frames of viral and human transcripts for RNA-seq and Ribo-seq samples. (G) Normalized P-site density in each of the reading frames of single- and dual-coded regions of M, NS, and PB1 segments of the influenza genome for RNA-seq and Ribo-seq samples for the +ifn +vir sample. (PDF) [file ppat.1007518.s006.pdf]

**A**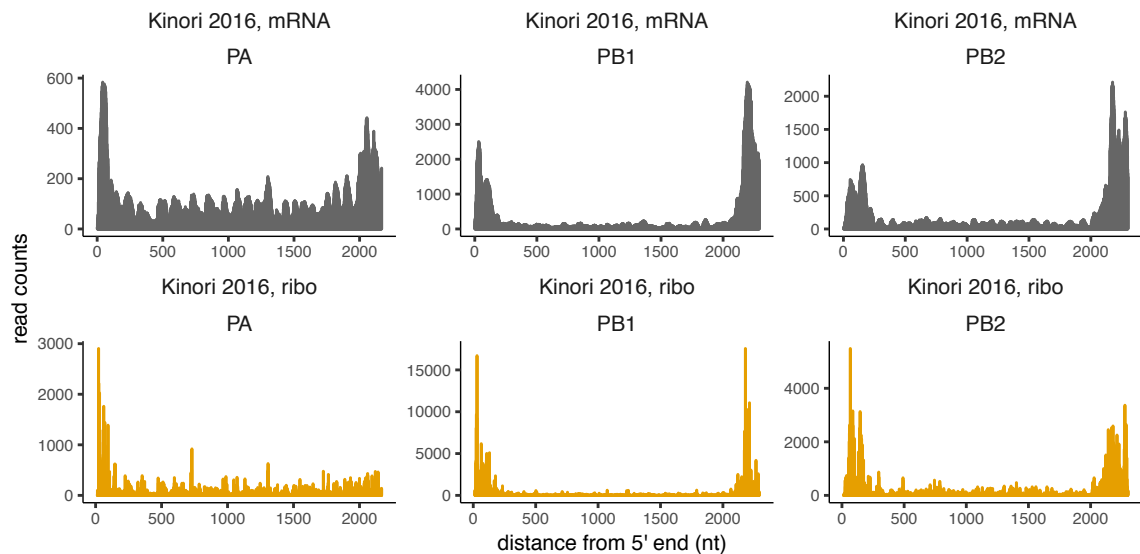**B**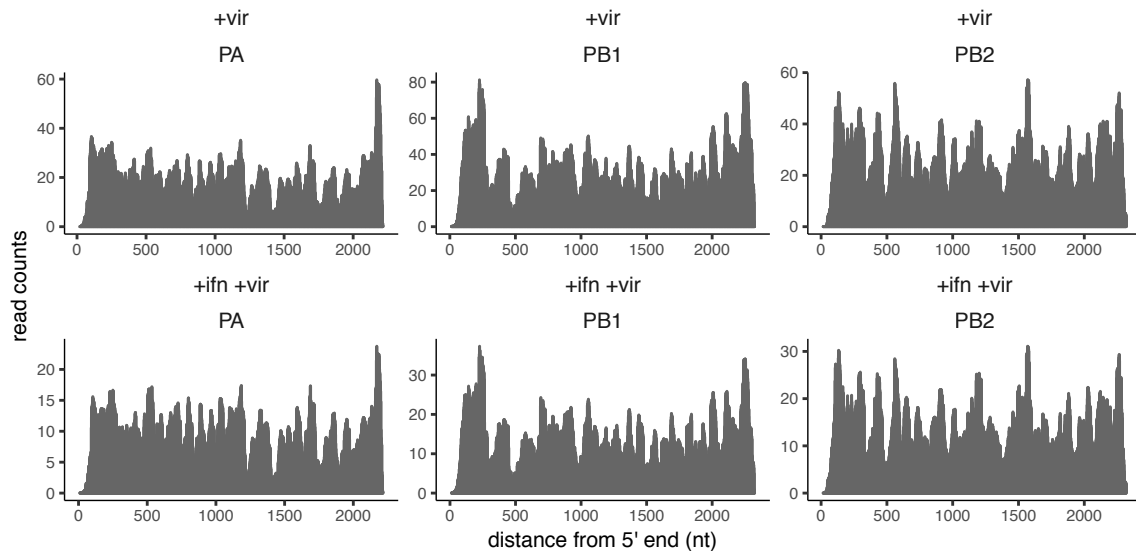

Supplement: S7 Fig — (A) Distribution of RNA-seq and Ribo-seq read density along the polymerase segments of influenza. Raw data from [30]. (B) Distribution of RNA-seq read density along the polymerase segments of influenza in our data. See Fig 3 for corresponding Ribo-seq density distribution. Defective viral particles are often characterized by the accumulation of large internal deletions in the polymerase segments. The large drop in coverage in panel A is consistent with the virus used in [30] containing a high burden of defective viral particles. (PDF) [file ppat.1007518.s007.pdf]

• ribo ▲ ribo + LTM

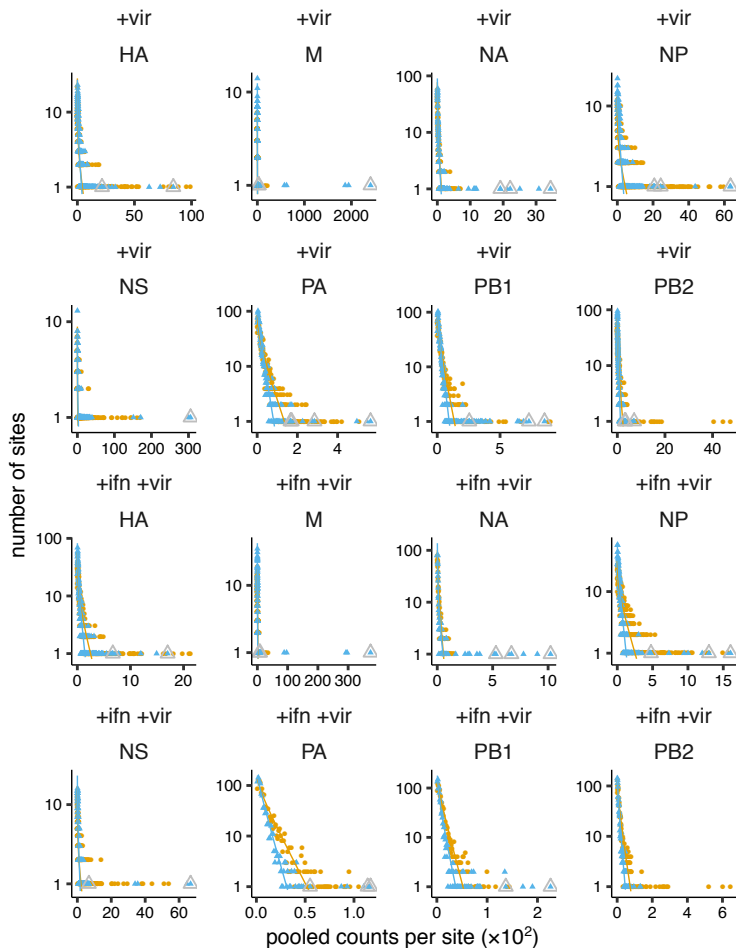

Supplement: S8 Fig — The background Ribo-seq and Ribo-seq + LTM counts for each influenza genome segment were fit to separate zero-truncated negative binomial distributions (shown as lines). The final called TIS are indicated by grey triangles. (PDF) [file ppat.1007518.s008.pdf]

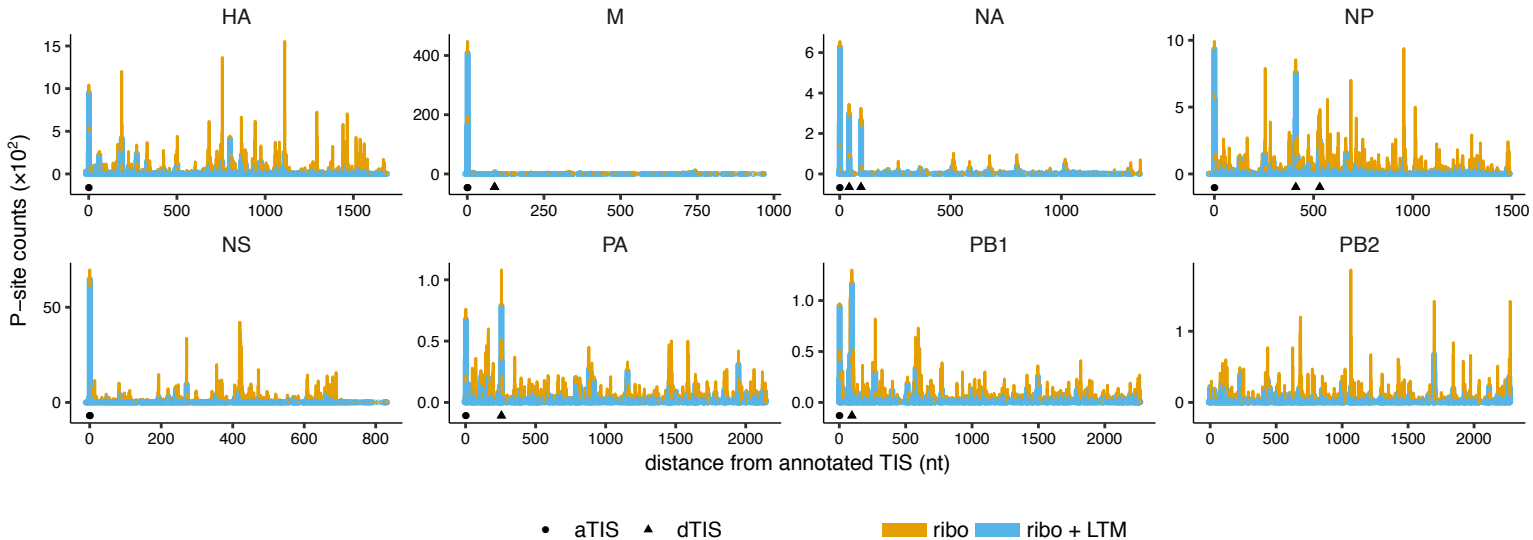

Supplement: S10 Fig — P-site counts from Ribo-seq and Ribo-seq + LTM assays are shown for all 8 influenza genome segments for our +ifn +vir sample. The counts from the two assays are shown as stacked bar graphs for ease of comparison. The candidate annotated TIS (circle) and downstream TIS (triangle) shared between the +vir and +ifn +vir samples are indicated below the coverage plots. (PDF) [file ppat.1007518.s010.pdf]

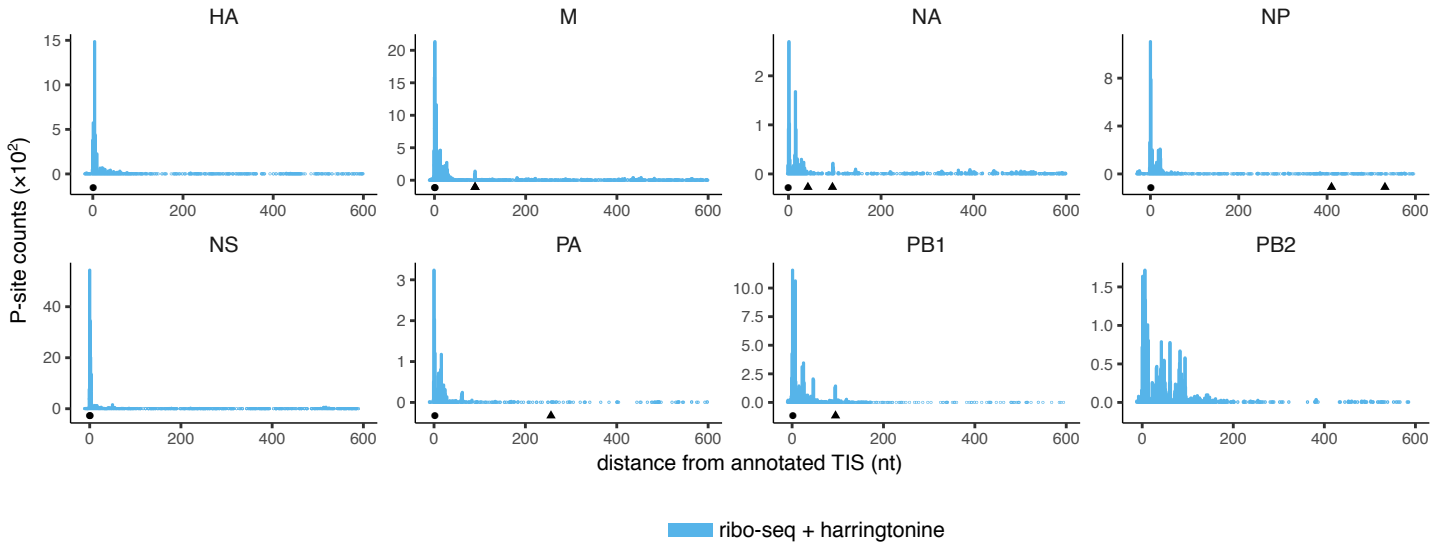

Supplement: S12 Fig — The P-site count coverage from [30] is overlaid with the 14 putative TIS identified in both of our +vir and +ifn +vir samples. Only the first 600 nucleotides of each gene sequence is shown. (PDF) [file ppat.1007518.s012.pdf]

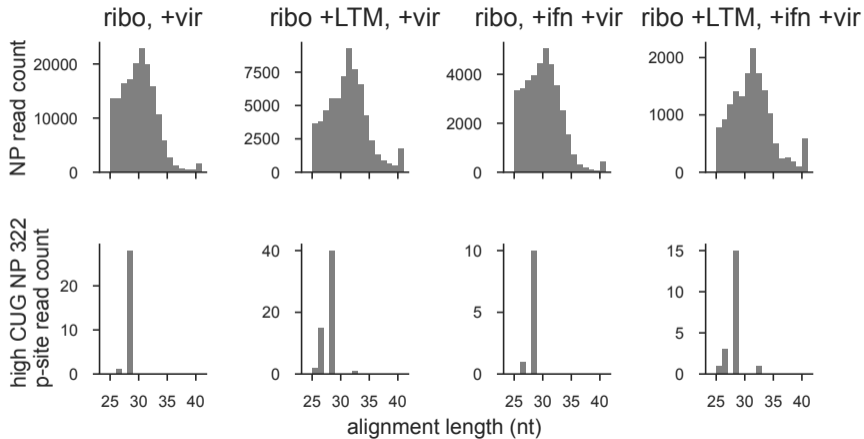

Supplement: S13 Fig — Distribution of alignment lengths for all NP reads and those with reads with P-site at position 322 of high CUG NP. (PDF) [file ppat.1007518.s013.pdf]

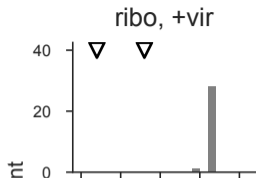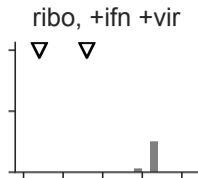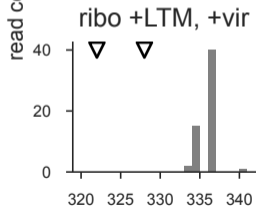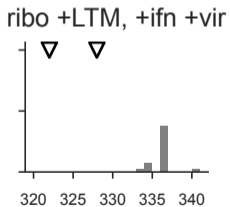

3' end of reads with p-site at position 322

Supplement: S14 Fig — For high CUG NP reads with P-site position at nucleotide 322 the distribution of 3′ end of alignment is shown. Recoded CUG codons at nucleotides 322 and 328 are indicated with triangles. (PDF) [file ppat.1007518.s014.pdf]

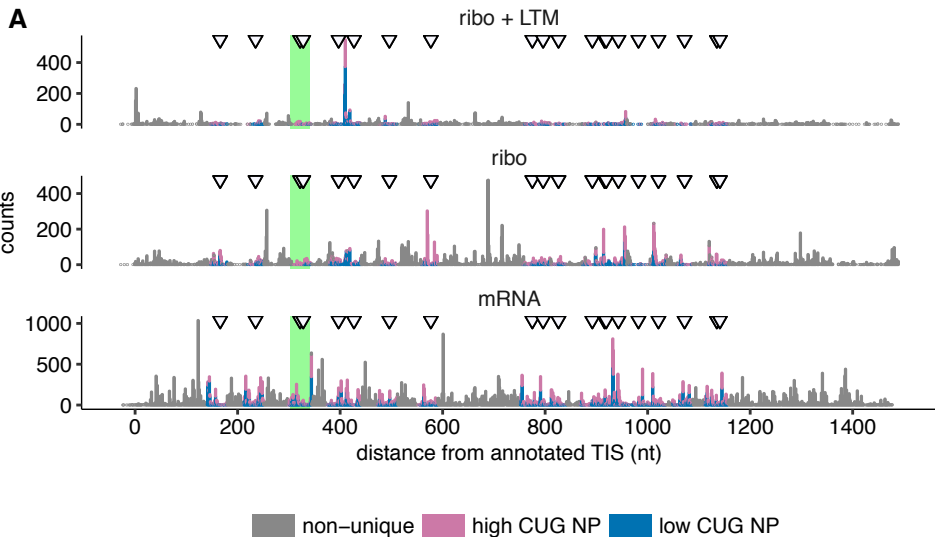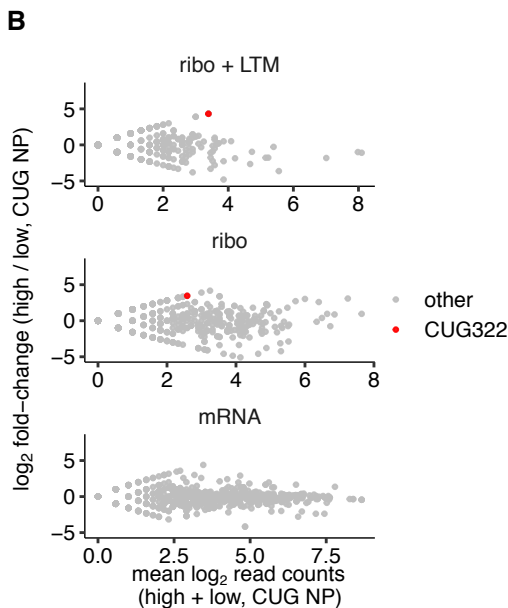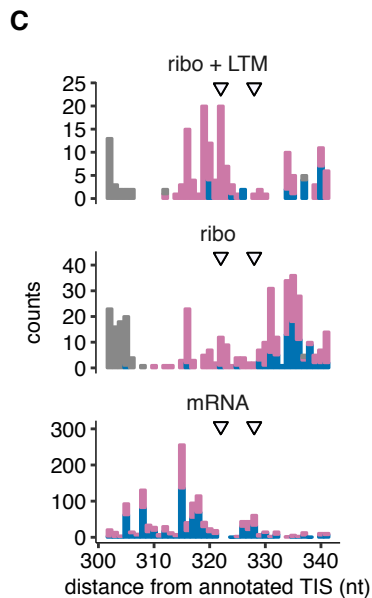

Supplement: S15 Fig — (A) Coverage of Ribo-seq + LTM, Ribo-seq, and RNA-seq reads that can be uniquely aligned to either the high CUG NP variant or the low CUG NP variant, and remaining non-unique reads. P-site counts are shown for Ribo-seq and Ribo-seq + LTM assays. 5′end counts are shown for RNA-Seq. Data are plotted as a stacked bar graph. Locations of the 20 CUG codons that are present in high CUG NP and synonymously mutated in low CUG NP are indicated by arrows. (B) The ratio of high CUG NP to low CUG NP coverage from A is plotted against their sum along the horizontal axis. There were no RNA-seq reads with counts at 322, so this point is not highlighted. (C) The green-highlighted region in A around the CUG322 codon is shown at greater horizontal magnification. See Fig 4 for +vir sample. (PDF) [file ppat.1007518.s015.pdf]

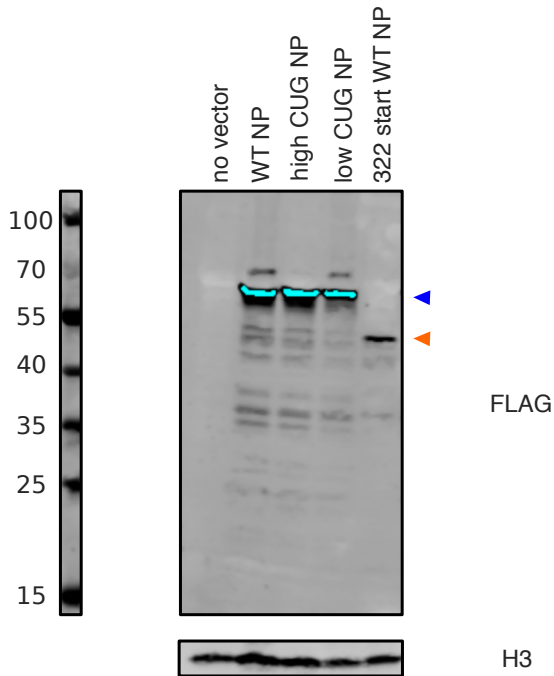

Supplement: S16 Fig — Western blot of 293T cells transfected with the indicated NP protein expression constructs. “322 start WT NP” is a size control construct that begins at nucleotide 322 of WT PR8 NP. Top panel: anti-Flag; bottom panel: anti-H3. Blue arrow corresponds to full length NP and orange arrow corresponds to expected size of NP fragment due to initiation at nucleotide 322. The blot was overexposed to sensitively detect truncated peptides, leading to saturation of the the full length NP band (shown in cyan). (PDF) [file ppat.1007518.s016.pdf]

**A**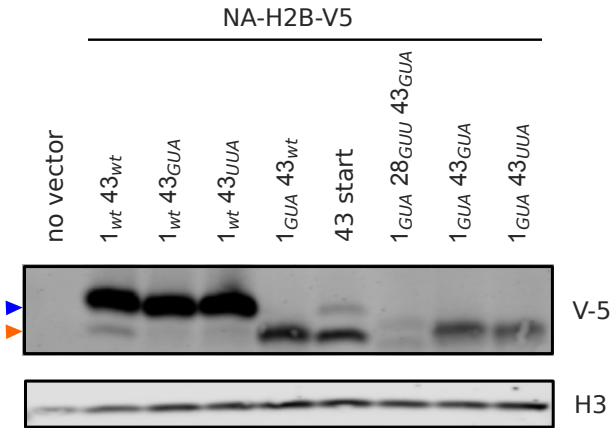**B**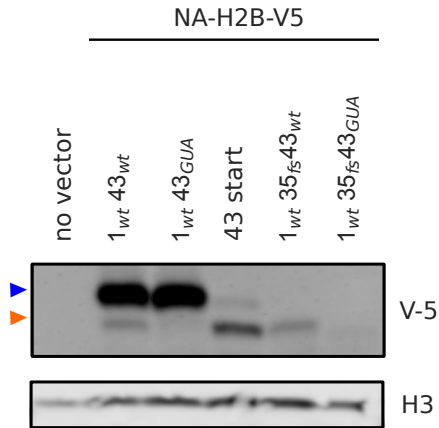

Supplement: S17 Fig — (A) and (B) Western blot of 293T cells transfected with NA-H2B-V5 constructs with mutations at the canonical or downstream start site. “43 start” is a size control construct that begins at site 43 of NA. 1GUA28GUU43GUA construct has a possible TIS (AUU codon) at position 28 mutated to GUU. 1GUA35fs43GUA construct has a U inserted at coding nucleotide 35, such that any initiation 5′ to the insert should not be detectable with the V5 antibody. Top panel: anti-V5 (blue arrow corresponds to full length NA and orange arrow corresponds to NA43); bottom panel: anti-H3. (PDF) [file ppat.1007518.s017.pdf]

**A**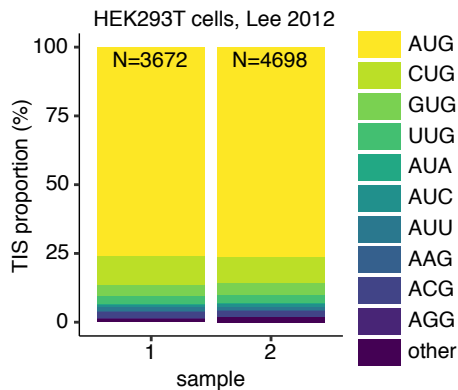**B**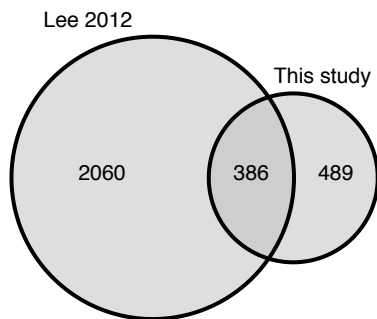**C**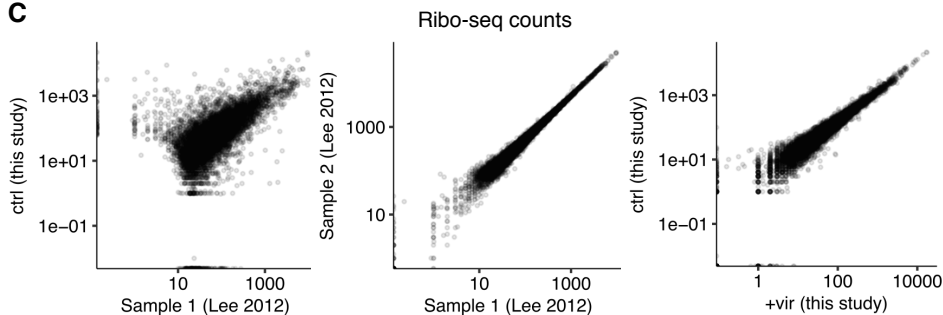**D**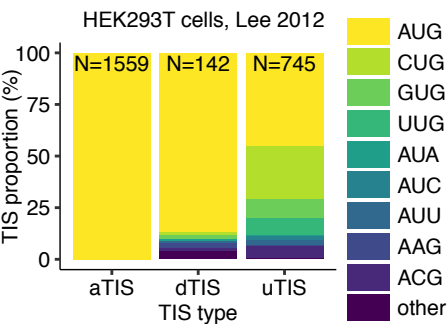**E**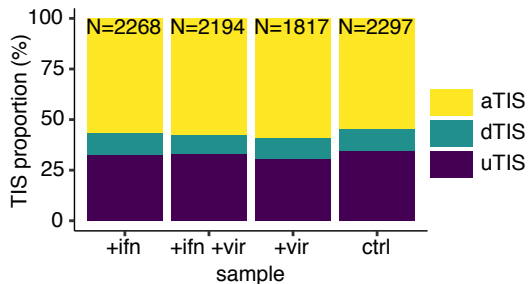**F**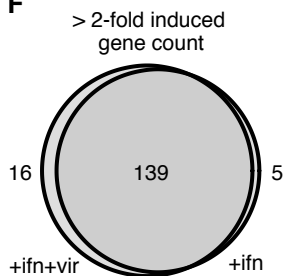

Supplement: S18 Fig — (A) Proportion of different near-cognate AUG codons (or other codons) overlapping with the called TIS in each of the two samples in [28]. N at the top of each bar indicates the total number of TIS called in each sample. (B) Overlap in high-confidence TIS between this study and Lee et al. [28]. High confidence TIS are the subset of TIS that are called across all samples in each study (2 in [28] and 4 in this study). (C) Comparison of Ribo-seq counts between samples in this study and from Lee 2012 [28]. Protein coding genes with at least 100 counts in one of the samples are plotted. (D) Proportion of different near-cognate AUG codons (or other codons) among the high-confidence TIS called in [28], stratified by TIS type. N at the top of each bar indicates the total number of high-confidence TIS of each type. (E) Proportion of different TIS types in each of the four samples used in this study. N at the top of each bar indicates the total number of TIS called in each sample. TIS not assigned to AUG or near-cognate AUG were excluded from this plot. (F) Overlap among the genes that are induced >2-fold upon either +ifn or +ifn +vir treatment with respect to the untreated sample. See Fig 6 for definition of induced genes. (PDF) [file ppat.1007518.s018.pdf]
