# Supplementary material for: Comprehensive profiling of translation initiation in influenza virus infected cells
Source: PLoS Pathog. 2019 Jan 23;15(1):e1007518. doi: 10.1371/journal.ppat.1007518 (PMC6361465; doi:10.1371/journal.ppat.1007518)
Supplement: S5 Fig — (A) Low CUG NP was generated by depleting wildtype PR8 NP of the common alternate start codons AUG, CUG, and GUG in all reading frames under constraints explained in Methods. High CUG NP was generated by adding 20 CUG codons into the low CUG NP background. All changes were synonymous with respect to reading frame 0. (B) Summary of the differences between wildtype PR8 NP (first number) and low CUG NP (second number) at the indicated codons. (PDF) [file ppat.1007518.s005.pdf]

A

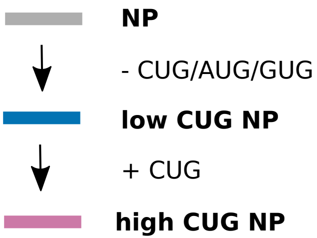

B

Codon	Reading frame 0	Reading frame 1	Reading frame 2
GUG	9 → 3	11 → 0	5 → 5
AUG	25 → 25	19 → 4	1 → 0
CUG	6 → 0	16 → 0	6 → 0
