# Supplementary material for: Comprehensive profiling of translation initiation in influenza virus infected cells
Source: PLoS Pathog. 2019 Jan 23;15(1):e1007518. doi: 10.1371/journal.ppat.1007518 (PMC6361465; doi:10.1371/journal.ppat.1007518)
Supplement: S9 Fig — (PDF) [file ppat.1007518.s009.pdf]

sample	codon	segment	frame	peptide length (aa)	distance to aTIS (nt)	ribo pval	LTM pval	called in
+ifn+vir	AUG	HA	0	565	0	2e-01	1e-14	both
+vir	AUG	HA	0	565	0	8e-02	0e+00	both
+ifn+vir	AUG	M	1	16	88	4e-01	8e-06	both
+vir	AUG	M	1	16	88	6e-01	7e-06	both
+ifn+vir	AUG	M	0	252	0	8e-14	0e+00	both
+vir	AUG	M	0	252	0	3e-14	0e+00	both
+ifn+vir	AUG	NA	2	2	95	1e-03	9e-09	both
+vir	AUG	NA	2	2	95	5e-05	0e+00	both
+ifn+vir	AUG	NA	0	453	0	5e-02	4e-15	both
+vir	AUG	NA	0	453	0	5e-02	0e+00	both
+ifn+vir	AUG	NA	0	439	42	1e-03	1e-10	both
+vir	AUG	NA	0	439	42	1e-04	0e+00	both
+ifn+vir	AUG	NP	0	498	0	3e-01	0e+00	both
+vir	AUG	NP	0	498	0	2e-01	0e+00	both
+ifn+vir	AUG	NP	0	362	408	7e-02	3e-14	both
+vir	AUG	NP	0	362	408	7e-02	3e-07	both
+ifn+vir	AUG	NS	0	230	0	1e-01	0e+00	both
+vir	AUG	NS	0	230	0	7e-02	0e+00	both
+ifn+vir	AUG	PA	0	716	0	3e-01	2e-08	both
+vir	AUG	PA	0	716	0	2e-01	2e-13	both
+ifn+vir	AUG	PA	0	631	255	1e-02	1e-08	both
+vir	AUG	PA	0	631	255	3e-03	3e-07	both
+ifn+vir	AUG	PB1	1	87	94	1e-01	2e-11	both
+vir	AUG	PB1	1	87	94	6e-02	0e+00	both
+ifn+vir	AUG	PB1	0	757	0	3e-01	2e-07	both
+vir	AUG	PB1	0	757	0	3e-01	2e-14	both
+vir	AUG	PA	1	1	1156	2e-01	1e-04	+vir
+vir	AUG	PB2	0	759	0	6e-01	2e-04	+vir
+ifn+vir	AUG	HA	2	7	800	2e-01	2e-06	+ifn+vir
+ifn+vir	AUG	M	0	5	741	5e-04	4e-08	+ifn+vir
+ifn+vir	AUG	NS	0	150	240	4e-01	3e-04	+ifn+vir
+ifn+vir	AUG	PA	2	17	113	4e-01	2e-04	+ifn+vir
+ifn+vir	other	NP	n.a.	n.a.	n.a.	9e-03	7e-06	both
+vir	other	NP	n.a.	n.a.	n.a.	5e-03	3e-06	both
+vir	other	HA	n.a.	n.a.	n.a.	5e-02	4e-05	+vir
+vir	other	M	n.a.	n.a.	n.a.	2e-01	1e-04	+vir
+vir	other	PA	n.a.	n.a.	n.a.	4e-01	1e-04	+vir
+vir	other	PB1	n.a.	n.a.	n.a.	4e-02	1e-05	+vir
+vir	other	PB2	n.a.	n.a.	n.a.	2e-01	6e-05	+vir
