# Supplementary material for: Comprehensive profiling of translation initiation in influenza virus infected cells
Source: PLoS Pathog. 2019 Jan 23;15(1):e1007518. doi: 10.1371/journal.ppat.1007518 (PMC6361465; doi:10.1371/journal.ppat.1007518)
Supplement: S11 Fig — Summary of the literature concerning alternate TIS in influenza. (PDF) [file ppat.1007518.s011.pdf]

influenza segment	protein	distance to aTIS (nt)	protein length (aa)	frame
PB1	PB1-F2 [14]	118	87	1
PB1	PB1-N40 [17]	141	718	0
PA	PA-N155 [20]	486	568	0
PA	PA-N182 [20]	567	535	0
M	M42 [21]	113	99	1
